# Value elicitation on a scenario of autonomous weapon system deployment: a qualitative study based on the value deliberation process

**DOI:** 10.1007/s43681-022-00211-2

**Published:** 2022-09-05

**Authors:** Ilse Verdiesen, Virginia Dignum

**Affiliations:** 1 Delft University of Technology, Delft, The Netherlands; 2 Umeå University, Umeå, Sweden

**Keywords:** Autonomous weapon systems, Value deliberation, Responsible AI, Accountability, Human oversight, Design for values

## Abstract

Ethical concerns on autonomous weapon systems (AWS) call for a process of human oversight to ensure accountability over targeting decisions and the use of force. To align the behavior of autonomous systems with human values and norms, the Design for Values approach can be used to consciously embody values in the deployment of AWS. One instrument for the elicitation of values during the design is participative deliberation. In this paper, we describe a participative deliberation method and results of a value elicitation by means of the value deliberation process for which we organized two panels each consisting of a mixture of experts in the field of AWS working in military operations, foreign policy, NGO’s and industry. The results of our qualitative study indicate not only that value discussion leads to changes in perception of the acceptability of alternatives, or options, in a scenario of AWS deployment, it also gives insight in to which values are deemed important and highlights that trust in the decision-making of an AWS is crucial.

## Introduction

According to the International Committee of the Red Cross (ICRC), ‘…certain types of autonomous weapons would cross legal and ethical lines.’ The ICRC mentioned this in their opening statement to the meeting of the Group of Governmental Experts (GGE) on emerging technologies in the area of Lethal Autonomous Weapons Systems (LAWS) of the Convention on Certain Conventional Weapons (CCW) of the United Nations in March 2022 [10]. The acute ethical concern of the ICRC is that an autonomous weapon system triggers a strike by itself which the user did not specify or of which they are not even aware. Ethical concerns are also raised by the International Panel on the Regulation of Autonomous Weapons (iPRAW) in their 2021 statement to the GGE LAWS [11]. The iPRAW continues to raise the question of whether—irrespective of the concrete consequences—life and death decisions should ever be transferred to machines and stressed that human dignity should be central in the use of force. In the 2022 GGE LAWS, the European Union stated that: ‘human beings must make the decisions with regard to the use of lethal force, exert control over lethal weapons systems that they use and remain accountable for decisions over the use of force to ensure compliance with International Law, in particular International Humanitarian Law’ [7].

**Fig. 1:**
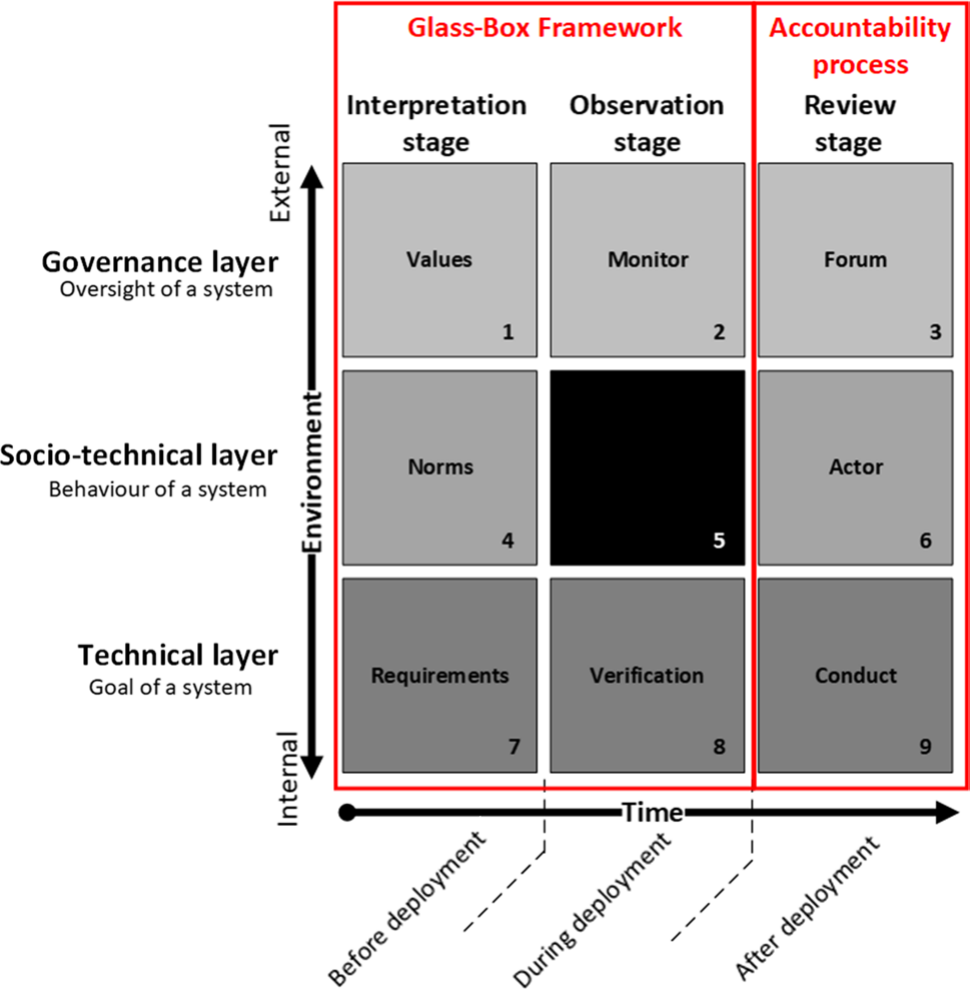
Glass box framework projected on CHOF (in Ref. [20])

These statements and ethical concerns call for a process of human oversight to ensure accountability over targeting decisions and the use of force. Accountability always requires strong mechanisms to oversee, discuss and verify the behavior of the system to check if its behavior is aligned with human values and norms [24]. Previous research by Verdiesen, Aler Tubella, and Dignum [20] operationalised human oversight over an autonomous system by proposing a socio-technical framework projecting the Glass Box approach on the Comprehensive Human Oversight Framework (CHOF) (see Fig. 1). The CHOF consists of three temporal phases—before, during and after deployment—and connects this with a governance, socio-technical and engineering perspective of control. The *governance* perspective of control describes which institutions or forums supervise the behavior of agents to govern their activities. The *socio-technical* perspective on control describes which agent (human or system) has the power to influence the behavior of another agent. In the *engineering* perspective on control, a mechanism compares the input and goal function of a system or device to the output by means of a feedback loop to take action to minimize the difference between outcome and goal [24]. The Glass Box approach [1] is a framework that focuses on the observable inputs and outputs of an intelligent system and can be used for monitoring adherence to the contextual interpretations of abstract values. The Glass Box approach consists of two phases which inform each other: (1) *interpretation* stage, which consists of a progressive process of concretising abstract values into specific design requirements and (2) *observation* stage, which is informed by the requirements on inputs and outputs identified in the interpretation stage, as they determine what must be verified and checked. Feedback between interpretation and observation stages throughout the lifespan of the system is necessary. By constraining the framework to observable elements of pre-deployment and post-deployment, one does not have to rely on assumptions based on the internal workings of the drone or the technical fluency of the operator.

Verdiesen, Aler Tubella, and Dignum’s research [20] presented an adaptation of the Glass Box approach for the inclusion of human oversight in autonomous drone deployment and demonstrated that it is possible to rely on observable elements without having to make assumptions made on the internal workings of the autonomous system or the technical fluency of the operator. This approach allows for a transparent human oversight process which ensures accountability when deploying an autonomous system. To align the behavior of a system with human values and norms, the Design for Values approach can be used to consciously embody values in the design of an AWS. One instrument for the elicitation of values during the design is participative deliberation. In this paper, we describe a qualitative study by means of a participative deliberation method and results of a value elicitation by means of the value deliberation process [15] for which we organized two panels each consisting of a mixture of experts in the field of AWS working in military operations, foreign policy, NGOs and industry. The results of the value deliberation process are reported in this paper. In Sect. 2, we first describe the background of the Comprehensive Human Oversight Framework and Glass Box Framework, next we describe values in general, followed by values related to AWS and then we discuss the value deliberation process as a method to conduct participative deliberation. In Sect. 3, we present our method by describing the research set-up, the scenario, the sample of the pilot and actual the study. In Sect. 4, we present the results on the ranking, the values that the participants selected and added to the pre-defined list and highlight trust in decision-making as one of the values that needs to be present when deploying an AWS. In Sect. 5, we conclude with a discussion on the implications of our findings, the limitations of our research and directions for further research.

## Background

In this section, we give a brief overview of relevant literature on the Comprehensive Human Oversight Framework and Glass Box Framework, values in general and related to AWS, and describe the value deliberation process that we used to elicit the values related to the deployment of AWS.

### Comphrensive human oversight framework

The Comprehensive Human Oversight Framework (CHOF) is a comprehensive approach on human oversight that goes beyond a singular engineering, socio-technical, or governance perspective on control. It connects an engineering, socio-technical, and governance perspective of control to three different temporal phases—before, during, and after deployment of an AWS. The *governance* perspective of control describes which institutions or forums supervise the behavior of agents to govern their activities. The *socio-technical* perspective on control describes which agent (human or system) has the power to influence the behavior of another agent. In the *engineering* perspective on control, a mechanism compares the input and goal function of a system or device to the output by means of a feedback loop to take action to minimize the difference between outcome and goal. These are depicted in the horizontal layers of Fig. 1. The vertical columns of the CHOF (x-axis) depict the three temporal phases: (1) before deployment of a system, (2) during deployment of a system, and (3) after deployment of a system. On the y-axis, the environment is plotted which ranges from more internal to more external to the technical system [23].

Verdiesen, Aler Tubella, and Dignum [20] presented an adaptation of the Glass Box approach for the inclusion of human oversight in autonomous drone deployment. The proposed framework includes an interpretation and an observation stage. During the interpretation stage of the Glass Box framework, values in the governance layer of the CHOF are turned into concrete norms before deployment of the autonomous system, constraining the observable elements and actions in the socio-technical layer of the CHOF, which in turn are translated into requirements in the technical layer of the CHOF. During deployment, the behavior and actions of an autonomous system are monitored in the governance layer and verified in the technical layer in the observation stage of the Glass Box framework. The block in the socio-technical layer during deployment is treated as a black box. A review stage is required after deployment as an accountability process in which a forum in the governance layer can hold an actor in the socio-technical layer accountable for its conduct in the technical layer. The outcome of the review stage should feed back into the interpretation stage for a next deployment of an autonomous system and thereby close the loop between the stages. The implementation concept in Ref. [20] to operationalise the socio-technical framework by projecting the Glass Box framework over the CHOF was based on existing operational norms within the Dutch Ministry of Defense, for example, rules of engagement, and left the value elicitation, which is the first step of the interpretation stage, out-of-scope for the implementation concept [20]. In this paper, we fill this gap by conducting value elicitation for the deployment of an AWS. For this purpose, we conducted the value deliberation process [15] for which we organized two panels each consisting of a mixture of experts in the field of AWS working in military operations, foreign policy, NGO’s and industry.

### Values

Values are a well-studied topic and many definitions can be found. Schwartz [17:p.21] states that values are ‘desirable trans-situational goals, varying in importance, that serve as guiding principles in the life of a person or other social entity.’ Friedman et al. [9] describe values more general and focus on what a person or group find important in life. This resembles the definition of Cheng and Fleischmann [3:p.2] in their meta-inventory of values: … ‘values serve as guiding principles of what people consider important in life’. Although a simple description, we adhere to this definition as we believe it captures the main characteristics of values best. Research on values related to AWS is a relatively new subject of study and values are often not explicitly mentioned in academic literature. Human Rights Watch mentions the lack of human emotion, accountability, responsibility, lack of human dignity and harm in their public reports [4, 5]. Responsibility, reduction of human harm, human dignity, honor and human sacrifice are mentioned by Johnson and Axinn [12] in the field of Military Ethics. Sharkey and Suchman [18] state that the values of accountability and responsibility are important to consider in the design of Robotic Systems for military operations. Asaro [2] refers to the principles of proportionality and discrimination which are, next to the principles of precaution, humanity and military necessity, captured in International Humanitarian Law. Previous work on values related to AWS, [21, 24] studied people’s perception on blame, trust, harm, human dignity, confidence, expectations, support, fairness and anxiety by comparing a scenario of the deployment of Human Operated drones to that of AWS. To select these values, we conducted a literature review, a short exploratory online survey and expert interviews [24]. The values selected to incorporate in the value deliberation process in this research are based on [21, 24] as we find this the most complete overview of values related to AWS.

### Value deliberation process

**Fig. 2:**
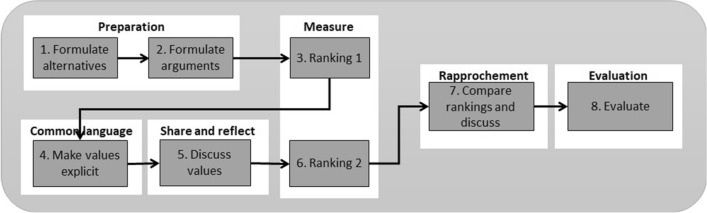
Phases of value deliberation process

Value deliberation is a form of participative deliberation aimed at creating mutual understanding on the various perspectives of the participants. By discussing values instead of solutions, a common ground and normative meta-consensus among stakeholders can be achieved [6]. Active participation in a debate offers the opportunity for people to develop and draft collective judgements on complex issues in real time. Deliberation will enhance critical thinking and reflection among its participants through a formalized and guided process. Through (online) deliberation, one can find solutions that consider and integrate various views on certain aspects of a topic. It enables people to learn about the different aspects of a complex (political) topic and to better understand each other’s positions [22]. Based on the practical implementation of deliberative democracy platforms, Fishkin [8] identifies five characteristics essential for legitimate deliberation: (1) information: accurate and relevant data are made available to all participants, (2) substantive balance: different positions are compared based on their supporting evidence, (3) diversity: all major positions relevant to the matter at hand and held by the public are considered, (4) conscientiousness: participants sincerely weigh all arguments, (5) equal consideration: views are weighed based on evidence, not on who is advocating a particular view. The value deliberation process that Pigmans [15] developed is inspired by the Delphi method. Where the Delphi method is designed to reach consensus between anonymous experts in an iterative process, the value deliberation process is aimed at reaching mutual understanding on the various stakeholder perspectives by direct interaction. The value deliberation process consists of six stages and eight steps (see Fig. 2).

Preparation is phase 1 in which the initiator briefs the topic and if applicable, the pre-defined solutions to the problem. Next, an independent facilitator takes over and starts with two preparatory steps in conjunction with the participants: step 1—*formulate alternatives* and step 2—*formulate arguments*. Phase 2 consists of measuring by *ranking* the alternatives from most preferable to least preferable (step 3). A Borda count is used to calculate the individual rankings. In phase 3, a common language is created by the *elicitation* of values (step 4). These values are *discussed* in phase 4 to create a mutual understanding (step 5). After a second *ranking* in step 6—based on the same principles in step 3—the rankings are *discussed* and compared to stimulate rapprochement in phase 5. The value deliberation process is concluded in phase 6 by an *evaluation* in which the participants reflect on the process and how it affected them. The five characteristics essential for legitimate deliberation of Fishkin [8] apply to the value deliberation process: (1) during the preparation phase, information and relevant data are distributed to all participants, (2) the steps to formulate the alternatives, arguments and conducting the value deliberation allow for comparing different positions and therefore provide substantive balance, (3) when inviting participants the initiator should ensure that the participants reflect all important perspectives so that diversity is reached, (4) an independent facilitator stresses the importance of conscientiously weighing all arguments, and (5) the facilitator should allow all participants to contribute to the discussion equally and underline that views are weighted on evidence and not on who proposes them. The value deliberation process meets Fishkin’s five characteristics for legitimate deliberation and therefore we applied it for the value elicitation of the interpretation stage of the Glass Box Framework.

## Methods

Value elicitation in the context of AWS deployment is qualitative research in which the participants interact and deliberate. The aim of the survey is to study if the value deliberation will change the participant’s perception on the acceptability of the alternatives regarding a scenario of AWS deployment. As the method for value elicitation, we chose the value deliberation process developed by Pigman [15] because it meets Fishkin’s five characteristics for legitimate deliberation and it was tested in a large-scale citizen’s summit event during the G1000 in July 2017 in Rotterdam [16].

### Research set-up

**Fig. 3:**
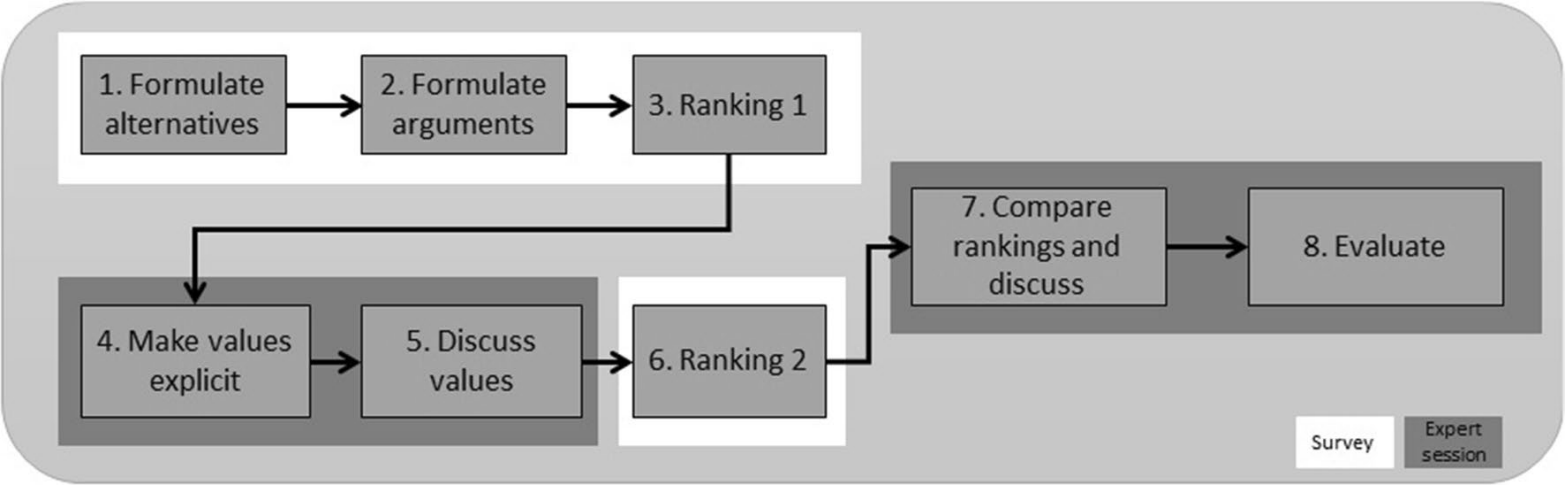
Value deliberation process differentiated in survey and expert online session (based on (Pigmans [15]))

Due to the COVID-19 restrictions, we designed an online value deliberation process instead of conducting the deliberation in person. We followed the process Pigmans [15] described (Fig. 3) and adjusted it to an online set-up consisting of a bipartite survey and a virtual session for the expert panel discussion. The first part of the survey was sent three days prior of the online discussion session and needed to be completed before the online session. The survey (see appendix) started with the scenario and the options (the *alternatives*) that the AWS could take were given (step 1 of Fig. 3). Next, the participants were asked to list an advantage and disadvantage (the *arguments*) for each option, which is step 2, and rank the options from most acceptable to least acceptable (*ranking 1*—step 3). During the online session, the second part of the survey was sent to guide the *value elicitation* (step 4). For each option, the participants were asked: *Which values are relevant for this option?* and *Are these values threatened or promoted in this option?* After filling in this part of the survey, the participants discussed values in the online session (step 5). Next, in step 6, the participants ranked the options a second time (*ranking 2*) in the survey. The online session concluded with a *comparison* and discussion on the ranking (step 7) and an *evaluation* (step 8). The advantage of the online setting is that the participants could join the survey from their own location which allowed for a diverse group with international participation without the need for traveling. The disadvantage of an online setting is that the non-verbal interaction and interpretation of facial expression is less than when conducting the session in person.

### Scenario and options

The following scenario was used throughout the survey to describe the situation:

*An autonomous weapon system provides force protection for soldiers that are clearing the road from improvised explosive devices. The autonomous weapon system is equipped with surveillance equipment, weapons (air-to-ground missiles) and flies autonomously in the Area of Operation. It is programmed to avoid flying over a restricted operating zone and an electronic warfare threat. The autonomous weapon system is equipped with facial and image recognition software for people, weapons and explosives. It is programmed with different options to engage when it recognizes a threat to the soldiers that are clearing the road. The autonomous weapon system detects movement behind a large rock near a narrow part of the road at a distance of 300 m of the road clearance soldiers.*

After reading this scenario, the participants read the options (the *alternatives*) each in turn and were asked to list an advantage and disadvantage per option (the *arguments*). The options presented to the participants are:

A: The autonomous weapon system identifies weapons aimed at the road clearing soldiers using image recognition. The autonomous weapon system warns the soldiers of the movement and takes no further action.
B: The autonomous weapon system identifies weapons aimed at the road clearing soldiers using image recognition. The autonomous weapon system asks permission to engage to neutralize the threat to the road clearance soldiers.
C: The autonomous weapon system identifies weapons aimed at the road clearing soldiers using image recognition. The autonomous weapon system automatically engages to neutralize the threat to the road clearance soldiers.
D: The autonomous weapon system positively identifies with a confidence of 99% using facial recognition all three persons sitting behind the rock as members of an opponent group aiming weapons at the road clearing soldiers. The autonomous weapon system automatically engages to neutralize the threat to the road clearance soldiers.
E: The autonomous weapon system positively identifies with a confidence of 99% using facial recognition all three persons sitting behind the rock as members of an opponent group aiming weapons at the road clearing soldiers. The autonomous weapon system shares the identification with the commander and asks permission to engage to neutralize the threat to the road clearance soldiers.
F : None of the options is acceptable.

### Sample pilot and actual study

A pilot study was conducted before the actual survey and online session was held. The aim of the pilot study was to improve the research set-up and if possible, the results could be used in the survey. Eight researchers (PhD students and post-docs) participated in the pilot but due to a flaw in the set-up—the two questionnaires could not be linked—the results were not usable. However, the pilot study gave valuable insight in the usability of this set-up for the value deliberation process and allowed us to correct the problem for the actual study. The actual study was held in two separate sessions. We sent 33 invitations to experts on AWS and 14 responded—a response rate of 42%. These experts were chosen based on their experience with, and knowledge of, autonomous systems. Most of them work or conduct research related to AWS or in a closely related adjacent field. We divided the 14 in two groups to ensure that the group was not too large for people to contribute to the online value discussion. The participants were a mix of military personnel (21%) and civilians (79%) working at the Dutch Ministry of Defense (25%), an NGO (8%), researchers (33%), policymakers (17%) and industry (17%). Session 1 consisted of six participants and resulted in 5 usable results, because one participant had not filled in the questionnaire before online session. Session 2 consisted of 8 participants and resulted in 7 usable results. One participant finished questionnaire before value discussion and therefore the value discussion was not of influence on the ranking of the options which impacted the research results. The total number of usable results is *n* = 12.We asked for some demographics; 93% of the participants has a university degree or PhD, 36% of the participants have worked with drones, 50% has worked with Artificial Intelligence and 36% has seen war or has been in a conflict zone.

The sample size (*n* = 12) is not uncommon in qualitative studies. Studies have found extreme variations in sample size in qualitative research studies across all research designs [14]. The sample size of a qualitative study can be determined by its *information power*. Information power depends on the aim of the study, sample specificity, use of established theory, quality of dialog and analysis strategy. Information power indicates that the more information the sample holds, relevant for the actual study, the lower number of participants is needed [13]. In our study, the panel consists of experts in the field of AWS deployment. The aim of the study is narrow, the experts have high specific knowledge on the topic, the theoretical background is sufficient, the quality of the dialog was strong and the analysis was done on a specific case (one scenario regarding the deployment of an AWS). The information power of our sample is high and therefore the sample size is sufficient. We use the results to explore the effect of value deliberation on the acceptability of options for AWS deployment to provide us with deeper insight into this real-world problem [19].

We chose not to increase the sample size by holding additional value deliberation sessions, because inviting laymen for this study will not provide additional qualitative data. In addition, we conducted an extra round of validation and invited four experts—who have not been part of the expert panel—to reflect on the results. Two experts responded and have reviewed the results and reflected on the usability for their field. Both experts indicated that the results are usable for their line of work and can apply the results in their work.

## Results

**Fig. 4:**
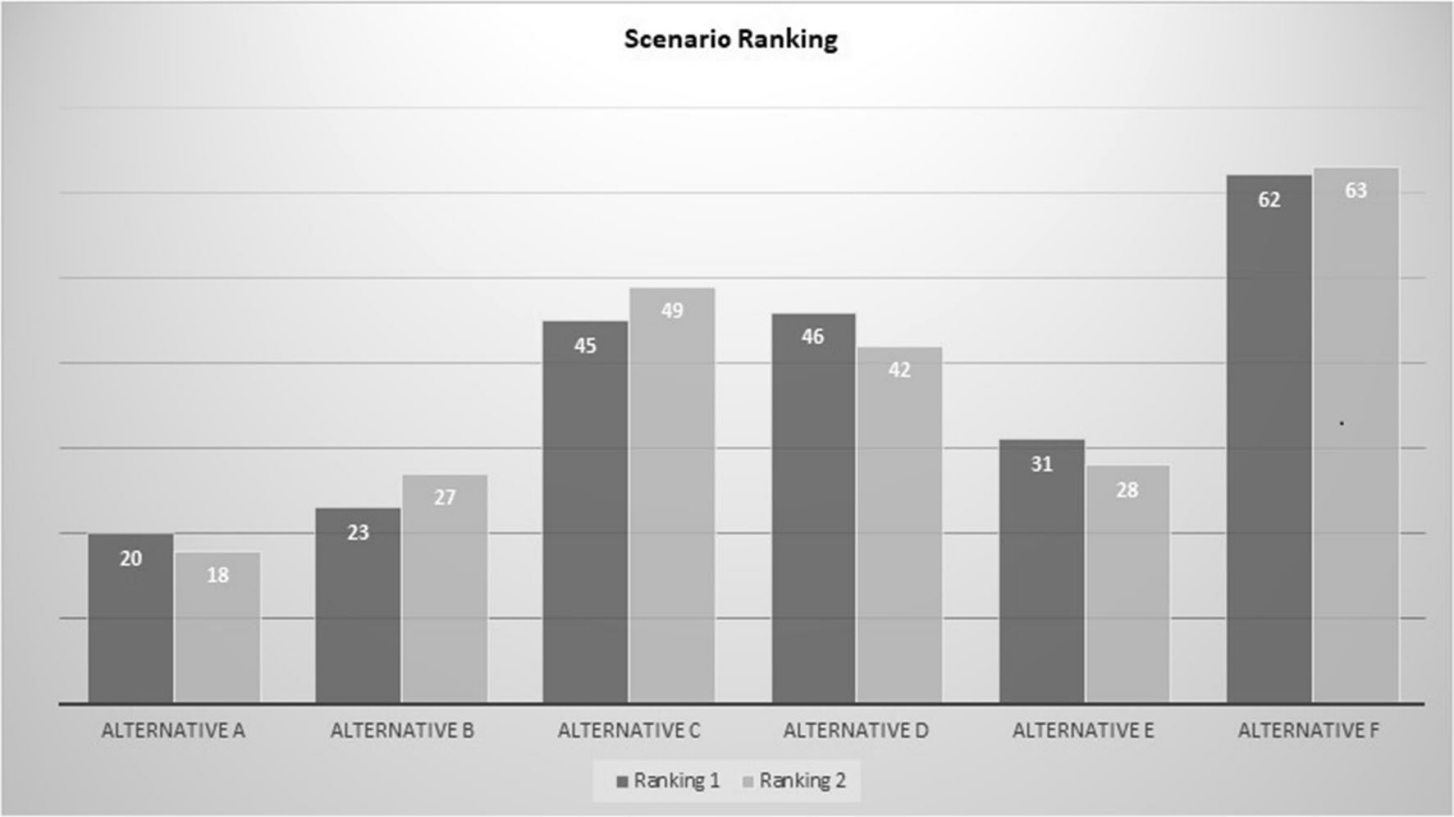
Overview results scenario ranking

The nature of the data is qualitative so no statistical techniques are applied to analyzed the results. The results are descriptive and are processed using the Ranking-Calculator from the Value Deliberation Toolbox (https://www.delftdesignforvalues.nl/valuedeliberation-toolbox/). The data were processed after the online session so the participants could not reflect on it during the session. The results in Fig. 4 show the ranking of the alternatives in round 1 (ranking 1 in Fig. 4) and round 2 (ranking 2 in Fig. 4). Ranking 1 is step 3 in the value deliberation process (Fig. 3) and ranking 2 is step 6. The alternative with the lowest score is the most acceptable alternative and the alternative with the highest score is the least acceptable. The order from most to least acceptable alternatives in round 1 is: A, B, E, C, D, F. In round 2, the order is: A, B, E, D, C, F. Based on the value deliberation between ranking 1 and 2, a change in the order of the acceptability of alternatives is noticeable. The acceptability of the alternative C and D is flipped in round 2 compared to round 1. Although a minor change, it is interesting because the participants were asked at the end of the value deliberation if they changed their ranking order. Some participants indicated to have consciously changed the order, but most participants replied that they did not, or did not intended to, leaving the option open that the value discussion could have influenced their ordering. One participant mentioned that the value discussion changed the way she read the options. Based on the results, it seems that some of the participants unconsciously changed the order of the acceptability of the alternatives.

Before the value deliberation, the participants were asked in part 2 of the survey for each of the alternatives: *which values are relevant for this option?* This is step 4—make values explicit- in the value deliberation process (Fig. 3). They could check a pre-defined list of the values: fairness, suffering, accountability, responsibility, safety, harm, human dignity, meaningful human control, predictability, privacy, trust, reliability, proportionality, blame, robustness, explainability. These values were selected based on [21] and the pilot study in which the participants indicated which values they missed in the pre-defined list. The values that were highlighted as relevant for the alternatives were: safety, meaningful human control, proportionality, accountability, responsibility, predictability, reliability and explainability. As part of the evaluation (step 8 in Fig. 3) participants were asked which values they missed on the pre-defined value list. Distinction, necessity, precaution, human autonomy, accuracy, human competences, relational and sociability between human and robot, mental and emotional health of the troops, usability and security were mentioned.

At the start of part 1 of the survey and before the first ranking, the participants were asked to list an advantage and disadvantage of each alternative (step 2 in Fig. 3). Early warning, safety of soldiers and quick response to threat were mentioned most as advantages. The disadvantages that were mentioned are: late response to threat, automation bias, false positives in identification and de-humanisation of the target. During the value deliberation (step 5 in Fig. 3), experts from different backgrounds discussed the context of the scenario and alternatives. Their experience and background determined how they viewed the scenario and alternatives and influenced their answer and ranking. For example, a scientist viewed the values as being part of the design process, for a policymaker, it was important that the system provides proper information and that the commander can review this information. One of the participants felt really uncomfortable with the image recognition and raised privacy issues in this ‘big brother’ scenario. An expert in computer vision viewed the 99% confidence as too uncertain, not reliable enough and not as an improvement of the system because it is more difficult to understand, but military personnel (nonexpert in computer vision) viewed the addition of 99% confidence as an increase in reliability of the system. Also, military personnel viewed the scenario based on the principles of the rules of engagement and hostile intent which gave context to the scenario to base their answers on. This shows that the difference in experience and background, for example technical expertise or operational experience, influences the answers and ranking of the participants in the value discussion. This can impact design choices that are based on value elicitation so the variety of participant’s background and level of expert knowledge should be taken into account when conducting the value deliberation and making design choices.

Another value that was discussed among the participants at the evaluation (step 8) was trust in the system. One participant stated that compared to human decision-making an AI system can make decisions with fewer errors than human decision-making (for example with Autonomous Vehicles). The option in which the AWS only was used as an early warning system was most acceptable and most trusted. Paraphrasing one of the military participants: “It is about understanding the strategy and context of the mission. We need to understand the impact of technology and our presence on the mission. We should think better of applying which technology in which context.’ This shows that not all applications of AWS in a mission context provide trust to military experts in the decision-making of the AWS. Human decision-making is in some cases more trusted and preferred. In general, the context in which an AWS is deployed impacts the meaning and weight people attribute to the values associated with the AWS.

## Conclusion and discussion

The value elicitation conducted using the value deliberation process not only shows that value discussion leads to changes in perception of the acceptability of alternatives in a scenario of AWS deployment, it also gives insight into which values are deemed important and highlights that trust in the decision-making of an AWS system is crucial. As a next step in the interpretation stage of the Glass Box framework, norms and requirements can be derived based on this value elicitation. These requirements will feed into the observation stage as observable elements to monitor and verify. The review stage is required after deployment as an accountability process of which findings should feed back into the interpretation stage for a next deployment of an autonomous system and thereby close the loop between the stages. This will be done in the next phase of our research. This qualitative study showed that based on the value deliberation between ranking 1 and 2 of the value deliberation process (Fig. 3), a change in the order of the acceptability of alternatives is noticeable (see Fig. 4). The acceptability of the alternative C and D is flipped in round 2 compared to round 1. Although it is a minor change it is interesting, because some participants indicated to have consciously changed the order, but most participants replied that they did not, or did not intended to, leaving the option open that the value discussion could have influenced their ordering. The values that were selected from the pre-defined list as relevant for the alternatives were safety, meaningful human control, proportionality, accountability, responsibility, predictability, reliability and explainability. Values that were missed on the pre-defined value list were distinction, necessity, precaution, human autonomy, accuracy, human competences, relational and sociability between human and robot, mental and emotional health of the troops, usability and security. The value discussion and evaluation disclosed that not all applications of AWS in a mission context provide trust to military experts in the decision-making of the AWS. Human decision-making is in some cases more trusted and preferred. In general, the context in which an AWS is deployed impacts the meaning and weight people attribute to the values associated with the AWS. The findings of this study imply that deliberate value discussion influences people perceptions of their values related to AWS. More general, active participation in a value discussion leads to a conscious, and sometimes unconscious, change in people’s preferences of alternatives. This could be beneficial in other areas than AWS for policy-making and citizen participation in local and national public administration. For example, to get citizen views on a municipal plan for the redevelopment of a local park or on a national level get input for water management policies. The application of the online value deliberation process method is not limited to AWS and can be used to other areas as well.

### Limitations and further research

Although the sample size of this qualitative survey is small (*n* = 12), the information power of our sample is high, because the aim of the study is narrow, the experts have high specific knowledge on the topic, the theoretical background is sufficient, the quality of the dialog was strong and the analysis was done on a specific case. Therefore, the sample size is sufficient to explore the effect of value deliberation on the acceptability of options for AWS deployment to provide us with deeper insight into this real-world problem. However, extending this study might be difficult, because there are not so many experts in the field of AWS with technical or military domain knowledge. As all the participants were military and technical experts in the field of AWS deployment, their domain knowledge influences the answers and ranking during the value discussion. Replicating the survey with non-experts might very well leads to different results and conclusions both on the ordering of alternatives as to the selection of relevant values. If this study is extended to a different field, for example to that of local or national policy development, the sample size needs to be determined based on the theory of information power for the context of that particular field and it might be that the sample size required for that context should be larger. A limitation is of this study is that the design of the value deliberation process in this study contains a survey in which the alternatives are already formulated as options at the start of the survey. This deviates from step 1 (see Fig. 3) of Pigman’s [15] model of the value deliberation process in which the formulation of the arguments is meant to created jointly a basic understanding of the alternatives. This deviation of step 1 prevents formulating additional alternatives and by this limits the understanding of the participants and could lead to alternative interpretations from the participants. Finally, a limitation of the value deliberation process itself is that the aim of the method is to get a mutual understanding of the different participant perspectives. However, the perspectives and opinions of the experts in the field of AWS deployment are very distinct both pros and cons AWS. During the value discussion, the participants gained more insight into each other’s perspectives, but a value deliberation discussion will not lead to mutual understanding among the participants—as is originally aimed with this method—on the arguments pro or con the deployment of AWS. As the debate on AWS is polarized, the discussion on AWS—mostly conducted in academic papers and in online blogs—is often one sided, e.g., either pros or cons, and a value deliberation can provide a balanced discussion on the topic and increase understanding. One way to conduct a balanced discussion on AWS by means of the value deliberation process could be to host sessions prior to the UN GGE LAWS to get input for the GGE. The online value deliberation process session would allow participants, providing that they have an internet connection, from over the world to discuss and give input on the subjects discussed at the UN in Geneva. By this, the discussion at the GGE LAWS will be enriched with other views and perspectives then those from the UN delegates alone.

In future work, we will close the feedback loop from the review stage as accountability process back to the interpretation stage of the Glass Box framework. This ensures that the lessons and recommendations from the review stage will be incorporated in the interpretation stage in a next iteration. We also will explore how the context in which an AWS is deployed impacts the meaning and weight people attribute to the values associated with AWS. It would be interesting to study if different contexts lead to a different set of relevant values. Another direction for further research is to apply the online value deliberation method we developed in this study to different domains to investigate the usefulness and applicability of this method to other topics to assess if it can lead to mutual understanding on values.

## Appendix: Questionnaire

### Survey on autonomous weapon systems part 1

Thank you for participating in this survey. The survey consists of 2 steps: (1) this online questionnaire and (2) an online discussion including a second questionnaire which will be held at a later time. This questionnaire is step 1 which should take about 20 min to complete. We will show you a theoretical scenario and options, ask you to identify an advantage and disadvantage per option and to rank the different options. Step 2 of the survey will be done by an online discussion for which you will receive a separate invitation. This survey is part of a Delft University of Technology scientific research project. Your decision to complete this survey is voluntary. The results of the research will be anonymized and may be presented at scientific meetings or published in scientific journals. Choosing the 'I agree' option on the bottom of this page indicates that you are at least 18 years of age and agree to complete this survey voluntarily. Please contact the researchers behind the study using the information below if you have any questions or concerns about the study.

Ilse Verdiesen e.p.verdiesen@tudelft.nl.

Virginia Dignum M.V.Dignum@tudelft.nl

**Do you agree to complete this survey voluntarily?**

**Please enter your personal code that was provided in the email invitation:**

**Scenario**

**Instruction**

Please read the scenario and options that correspond with the scenario. After reading the scenario and options, list an advantage and disadvantage per option.

**Scenario**

*An autonomous weapon system provides force protection for soldiers that are clearing the road from improvised explosive devices. The autonomous weapon system is equipped with surveillance equipment, weapons (air-to-ground missiles) and flies autonomously in the Area of Operation. It is programmed to avoid flying over a restricted operating zone and an electronic warfare threat. The autonomous weapon system is equipped with facial and image recognition software for people, weapons and explosives. It is programmed with different options to engage when it recognizes a threat to the soldiers that are clearing the road. The autonomous weapon system detects movement behind a large rock near a narrow part of the road at a distance of 300 m of the road clearance soldiers.*

**Option A:** The autonomous weapon system identifies weapons aimed at the road clearing soldiers using image recognition. The autonomous weapon system warns the soldiers of the movement and takes no further action.

**Advantage:**

**Disadvantage:**

**Option B:** The autonomous weapon system identifies weapons aimed at the road clearing soldiers using image recognition. The autonomous weapon system asks permission to engage to neutralize the threat to the road clearance soldiers.

**Advantage:**

**Disadvantage:**

**Option C:** The autonomous weapon system identifies weapons aimed at the road clearing soldiers using image recognition. The autonomous weapon system automatically engages to neutralize the threat to the road clearance soldiers.

**Advantage:**

**Disadvantage:**

**Option D:** The autonomous weapon system positively identifies with a confidence of 99% using facial recognition all three persons sitting behind the rock as members of an opponent group aiming weapons at the road clearing soldiers. The autonomous weapon system automatically engages to neutralize the threat to the road clearance soldiers.

**Advantage:**

**Disadvantage:**

**Option E:** The autonomous weapon system positively identifies with a confidence of 99% using facial recognition all three persons sitting behind the rock as members of an opponent group aiming weapons at the road clearing soldiers. The autonomous weapon system shares the identification with the commander and asks permission to engage to neutralize the threat to the road clearance soldiers.

**Advantage:**

**Disadvantage:**

**Ranking options**

**Instruction**

Please read the scenario and options that correspond with the scenario and rank the options from most acceptable to least acceptable.

**Scenario**

An autonomous weapon system provides force protection for soldiers that are clearing the road from improvised explosive devices. The autonomous weapon system is equipped with surveillance equipment, weapons (air-to-ground missiles) and flies autonomously in the Area of Operation. It is programmed to avoid flying over a restricted operating zone and an electronic warfare threat. The autonomous weapon system is equipped with facial and image recognition software for people, weapons and explosives. It is programmed with different options to engage when it recognizes a threat to the soldiers that are clearing the road. The autonomous weapon system detects movement behind a large rock near a narrow part of the road at a distance of 300 m of the road clearance soldiers.

**Please rank the following options from most acceptable to least acceptable.**

1. A: The autonomous weapon system identifies weapons aimed at the road clearing soldiers using image recognition. The autonomous weapon system warns the soldiers of the movement and takes no further action.
2. B: The autonomous weapon system identifies weapons aimed at the road clearing soldiers using image recognition. The autonomous weapon system asks permission to engage to neutralize the threat to the road clearance soldiers.
3. E: The autonomous weapon system positively identifies with a confidence of 99% using facial recognition all three persons sitting behind the rock as members of an opponent group aiming weapons at the road clearing soldiers. The autonomous weapon system shares the identification with the commander and asks permission to engage to neutralize the threat to the road clearance soldiers.
4. D: The autonomous weapon system positively identifies with a confidence of 99% using facial recognition all three persons sitting behind the rock as members of an opponent group aiming weapons at the road clearing soldiers. The autonomous weapon system automatically engages to neutralize the threat to the road clearance soldiers.
5. C: The autonomous weapon system identifies weapons aimed at the road clearing soldiers using image recognition. The autonomous weapon system automatically engages to neutralize the threat to the road clearance soldiers.
6. F: None of the options is acceptable.

**Background information**

We thank you for your time spent taking this survey. Please press **send** to record your response.

### Survey on autonomous weapon systems part 2

Thank you for participating in this survey. The survey consists of 2 steps: (1) an online questionnaire which was already send and (2) an online discussion including this second questionnaire.

This questionnaire is part of step 2. We will show you a theoretical scenario, a list of values and ask you which values are important in the scenario. Next, we will discuss in the online video call why these values are important and ask you to rank the different options.

This survey is part of a Delft University of Technology scientific research project. Your decision to complete this survey is voluntary. The results of the research will be anonymized and may be presented at scientific meetings or published in scientific journals. Choosing the ‘I agree’ option on the bottom of this page indicates that you are at least 18 years of age and agree to complete this survey voluntarily.

Please contact the researchers behind the study using the information below if you have any questions or concerns about the study.

Ilse Verdiesen e.p.verdiesen@tudelft.nl.

Virginia Dignum M.V.Dignum@tudelft.nl.

**Do you agree to complete this survey voluntarily?**

**Please enter your personal code that you received in the email invitation:**

**Scenario**

**Instruction**

Please read the scenario*, the list of values and options that correspond with the scenario. Next, describe which values from the list you believe are relevant for the options.

* The scenario is the same as in part one of the survey.

**Scenario**

*An Autonomous Weapon System provides force protection for soldiers that are clearing the road from improvised explosive devices. The Autonomous Weapon System is equipped with surveillance equipment, weapons (air-to-ground missiles) and flies autonomously in the Area of Operation. It is programmed to avoid flying over a restricted operating zone and an electronic warfare threat. The Autonomous Weapon System is equipped with facial and image recognition software for people, weapons and explosives. It is programmed with different options to engage when it recognizes a threat to the soldiers that are clearing the road. The Autonomous Weapon System detects movement behind a large rock near a narrow part of the road at a distance of 300 m of the road clearance soldiers.*

**List of values**

- Fairness
- Suffering
- Accountability
- Responsibility
- Safety
- Harm
- Human dignity
- Meaningful human control
- Predictability
- Privacy
- Trust
- Reliability
- Proportionality
- Blame
- Robustness
- Explainability

**Option A*:** The Autonomous Weapon System identifies weapons aimed at the road clearing soldiers using image recognition. The Autonomous Weapon System warns the soldiers of the movement and takes no further action.

*All the options are the same as in part one of the survey.

**Which values are relevant for this option?**

**Are these values threatened or promoted in this option?**

**Option B*:** The Autonomous Weapon System identifies weapons aimed at the road clearing soldiers using image recognition. The Autonomous Weapon System asks permission to engage to neutralize the threat to the road clearance soldiers.

*All the options are the same as in part one of the survey.

**Which values are relevant for this option?**

**Are these values threatened or promoted in this option?**

**Option C*:** The Autonomous Weapon System identifies weapons aimed at the road clearing soldiers using image recognition. The Autonomous Weapon System automatically engages to neutralize the threat to the road clearance soldiers.

*All the options are the same as in part one of the survey.

**Which values are relevant for this option?**

**Are these values threatened or promoted in this option?**

**Option D*:** The Autonomous Weapon System positively identifies with a confidence of 99% using facial recognition all three persons sitting behind the rock as members of an opponent group aiming weapons at the road clearing soldiers. The Autonomous Weapon System automatically engages to neutralize the threat to the road clearance soldiers.

*All the options are the same as in part one of the survey.

**Which values are relevant for this option?**

**Are these values threatened or promoted in this option?**

**Option E*:** The Autonomous Weapon System positively identifies with a confidence of 99% using facial recognition all three persons sitting behind the rock as members of an opponent group aiming weapons at the road clearing soldiers. The Autonomous Weapon System shares the identification with the commander and asks permission to engage to neutralize the threat to the road clearance soldiers.

*All the options are the same as in part one of the survey.

**Which values are relevant for this option?**

**Are these values threatened or promoted in this option?**

**Discussion values**

Please return to the online session to discuss which values you listed as relevant for the different options.

After the discussion we will continue with the survey.

**Ranking options**

**Instruction**

Please read the scenario* and options that correspond with the scenario and rank the options from most acceptable to least acceptable.

* The scenario is the same as in part one of the survey.

**Scenario**

*An Autonomous Weapon System provides force protection for soldiers that are clearing the road from improvised explosive devices. The Autonomous Weapon System is equipped with surveillance equipment, weapons (air-to-ground missiles) and flies autonomously in the Area of Operation. It is programmed to avoid flying over a restricted operating zone and an electronic warfare threat. The Autonomous Weapon System is equipped with facial and image recognition software for people, weapons and explosives. It is programmed with different options to engage when it recognizes a threat to the soldiers that are clearing the road. The Autonomous Weapon System detects movement behind a large rock near a narrow part of the road at a distance of 300 m of the road clearance soldiers.*

**Please rank the following options from most acceptable to least acceptable.**

1. A: The Autonomous Weapon System identifies weapons aimed at the road clearing soldiers using image recognition. The Autonomous Weapon System warns the soldiers of the movement and takes no further action.
2. B: The Autonomous Weapon System identifies weapons aimed at the road clearing soldiers using image recognition. The Autonomous Weapon System asks permission to engage to neutralize the threat to the road clearance soldiers.
3. E: The Autonomous Weapon System positively identifies with a confidence of 99% using facial recognition all three persons sitting behind the rock as members of an opponent group aiming weapons at the road clearing soldiers. The Autonomous Weapon System shares the identification with the commander and asks permission to engage to neutralize the threat to the road clearance soldiers.
4. D: The Autonomous Weapon System positively identifies with a confidence of 99% using facial recognition all three persons sitting behind the rock as members of an opponent group aiming weapons at the road clearing soldiers. The Autonomous Weapon System automatically engages to neutralize the threat to the road clearance soldiers.
5. C: The Autonomous Weapon System identifies weapons aimed at the road clearing soldiers using image recognition. The Autonomous Weapon System automatically engages to neutralize the threat to the road clearance soldiers.
6. F: None of the options is acceptable.

**Discussion options**

Please return to the online session to discuss the ranking of the options.

After the discussion we will continue with the survey.

**Background information**

We thank you for your time spent taking this survey. Please press **send** to record your response.
